# Perception of content and non-content expert facilitators of PBL according to students’ performance levels

**DOI:** 10.12669/pjms.316.8691

**Published:** 2015

**Authors:** Noor Akmal Shareela Ismail, Ekram Alias, Khaizurin Tajul Arifin, Mohd Hanafi A Damanhuri, Norwahidah Abd Karim, Goon Jo Aan

**Affiliations:** 1 Dr. Noor Akmal Shareela Ismail, PhD. Department of Biochemistry, Faculty of Medicine, University Kebangsaan Malaysia, Jalan Yaacob Latif, 56000 Kuala Lumpur, Malaysia; 2 Dr. Ekram Alias, PhD. Department of Biochemistry, Faculty of Medicine, University Kebangsaan Malaysia, Jalan Yaacob Latif, 56000 Kuala Lumpur, Malaysia; 3 Dr. Khaizurin Tajul Arifin, PhD. Department of Biochemistry, Faculty of Medicine, University Kebangsaan Malaysia, Jalan Yaacob Latif, 56000 Kuala Lumpur, Malaysia; 4 Dr. Mohd Hanafi A Damanhuri, PhD. Department of Biochemistry, Faculty of Medicine, University Kebangsaan Malaysia, Jalan Yaacob Latif, 56000 Kuala Lumpur, Malaysia; 5 Dr. Norwahidah Abd Karim, PhD. Department of Biochemistry, Faculty of Medicine, University Kebangsaan Malaysia, Jalan Yaacob Latif, 56000 Kuala Lumpur, Malaysia; 6 Dr. Goon Jo Aan, PhD. Department of Biochemistry, Faculty of Medicine, University Kebangsaan Malaysia, Jalan Yaacob Latif, 56000 Kuala Lumpur, Malaysia

**Keywords:** Problem-based learning, Medical Biochemistry, Undergraduate students, Facilitator, Content expert, Meet-the-expert-session

## Abstract

**Objective:**

Problem-based learning (PBL) is a student-centred learning system that involves multidisciplinary fields focused on problem solving. Facilitators of PBL are not necessarily content experts but little is known on how this concept has affected the outcomes of PBL sessions in learning Medical Biochemistry. We aimed to evaluate the impact of having the content expert as a facilitator in conducting PBL.

**Methods:**

A total of 150 first and second year medical students from the University Kebangsaan Malaysia were interviewed with a validated set of questions to acquire their views on the roles of facilitators in PBL in learning Medical Biochemistry. Their achievement were evaluated through their essay marks derived from various PBL packages.

**Results:**

All respondents agreed that PBL sessions associated with Medical Biochemistry are best appreciated when conducted by a content-expert facilitator. Their exam marks reflected well on their perception.

**Conclusion:**

PBL sessions related to Medical Biochemistry is best facilitated by Biochemistry lecturers as the content experts.

## INTRODUCTION

Problem-Based Learning (PBL) is a student-centred learning introduced in the late 1960s to develop problem solving skills as well as to instil lifelong learning among the students.^1^ It challenges students to learn through engagement in a real case scenario.^2^ PBL also aims to simultaneously develop both problem solving strategies and interdisciplinary knowledge bases and skills by placing students in the active role of problem-solvers, confronted with ill-structured situations that simulates similar problems that they are likely to face as future doctors.^3^ PBL in medicine is a teaching-learning method integrating basic science and clinical setting partially serves as the substitutes of the conventional methods. PBL packages are designed to integrate the knowledge of medical sciences in problem solving.^4^,^5^ The processes involved such as problem recognition, hypothesis generation and formulation of learning issues leads to development of PBL outcomes. These generic skills include the ability to apply knowledge in problem solving, team work, communication skills, professionalism, leadership and critical thinking.^4^,^5^

In University Kebangsaan Malaysia (UKM), PBL is conducted as a part of the integrated system-based curriculum in preclinical that involves different disciplines: Biochemistry, Anatomy, Pharmacology, Physiology, Pathology and Microbiology. A group of 10 to 12 students are facilitated by lecturers from different departments in the Faculty of Medicine. PBL sessions are done in two separate days with more than one day of interval between the sessions to enable the students to perform thorough search and reading regarding the learning issues formulated from discussion in the first PBL session. Gathered information is discussed, analysed and summarised by the students in the second PBL session to resolve the learning issues. Lastly, a concept map is constructed by the group of students to summarise the case involved. At the end of the session, a self-reflection on individual performances in PBL is carried out by the students. The performance of students including their ability to hypothesise, explain, discuss and search for information is assessed by facilitators using a standard scoring sheet prepared by the Department of Medical Education, UKM.

Each of the PBL components plays a crucial role in order to meet the objectives to develop an effective reasoning process, self-directed learning skills, increase motivation for lifelong learning, interpersonal and communication skills thus to connect new information to previous knowledge^7^. In addition, as part of fulfilling the objectives of PBL in promoting self-directed learning, contribution from the facilitators towards the group discussion is expected to be very minimal. The role of facilitator has been well described as the teacher plays an important role as a facilitator to ask critical questions and explain new knowledge and give insights in problem solving.^8^ For that purpose, there are opinions discouraging facilitators from among topic content experts. To date, there is no medical faculty that has conducted PBL solely using the content expert. Revolving on the facilitators as our main concern in enabling the students to reach the learning outcome, this study was designed to explore the perception of UKM medical students towards the necessity of having content experts as their facilitators during PBL as compared to the non-content facilitator, as underlined in the principle of PBL and to evaluate the effectiveness of having the content expert to their examination marks respectively.

## METHODS

### Study Design and study settings

This is a cross-sectional survey design using a sample of first year of Medical students. We carried out a series of interviews on two cohorts of separate academic years, 2011-2012 and 2012-2013. A total of 150 preclinical students in Faculty of Medicine, UKM were involved in this study. A random selection of students was made from both cohorts for this study. They were assigned into groups of 10 students for an interview to get their views on the efficacy of PBL and their thoughts on the role of facilitators in PBL in learning Biochemistry. Students were asked with a set of open-ended questions, which has been validated by two independent experts, regarding the effectiveness of having content experts as facilitator in PBL as a part of their teaching learning method. The instrument has been tested for reliability through a pilot study conducted with 12 medical students in UKM previously. Each of interview sessions was facilitated and recorded (audio/visual) by a facilitator. Students who could not attend the interview session were given a similar set of questions online and answers were captured through email. Prior to the interview session, participating students were provided with a verbal and written consent form before commencing on this study. We then tracked their performance in essay marks that corresponds well to the cases discussed in the PBL previously. Students were categorized into three different groups of CGPA, 1) CGPA: 3.50- 4.00 (excellent), 2) CGPA: 3.00-3.49 (good) and 3) CGPA 2.00-3.00 (average).

### Statistical Analysis

Data were analysed using SPSS version 20. Results of descriptive analysis were tabulated in the form of mean and standard deviation. The differences between groups in each category of CGPA were tested using Student’s t-test (p<0.05) and later signified by asterisks in the table.

## RESULTS

The demography of the respondents are listed in Table-I. All the participants reflected of Malay (65%), Chinese (16%) and Indian (19%) which represent the sample population of the preclinical students in UKM. We tried to recruit an equal number between genders in our study however, the cohort in the preclinical years comprises of more female students (57.3%) as compared to male (42.6%). The data and outcomes of the interviews were summarised and outlined as below:

**Table-I:** Demographic of the respondents in Faculty of Medicine, University Kebangsaan Malaysia, Malaysia.

Variables	Frequency (n)
*Age cohorts*	
21 and below	75
Above 21 years	75
*Gender*	
Male	64
Female	84
*Race*	
Malay	98
Chinese	24
Indian	28
*CGPA*	
3.00-4.00 (excellent)	50
2.50-2.99 (good)	50
2.00-2.49 (average)	50

### PBL as a preferred teaching learning method in UKM

Students were first asked whether they appreciated PBL as a part of the teaching learning method in learning Medical Biochemistry. Fifty percent of the respondents agreed that PBL was their favourite as compared to other teaching methods. Two third of the cohorts agreed that PBL involved a lot of clinical cases that were significant to their understanding towards each topic. Hundred percent of our respondents were well informed how PBL is implemented. Over 75% of them preferred session 2 as compared to session 1 depending on the case given. Among these, they responded they will get less interested if the facilitators were not probing them to get the right learning issues. This is well portrayed by these snippets:

***“Biochemistry involves a lot of pathways and a bit complicated. The non-expert facilitators don’t know how to guide us to understand that”***

***“The (non-expert) facilitators don’t know how to probe us in getting the right learning issues and I think because they’re not the content expert”***

***“Each time we’re presenting our learning issues, there will be no response from the non-content expert facilitators. So, we don’t know whether the information presented is right or wrong”***

### Content expert vs non-content expert as a facilitator

Based on the transcripts of the interview, participation of the non-content expert facilitators was seen lesser in session two as compared to session one. However, all respondents agreed that they will anticipate more outcomes from the case in which the facilitator is the content expert. This is because the content expert will inquire more on their knowledge towards the subject. The probing makes them well prepared ahead of time especially in session 2. The summary is shown as in the excerpts:

***“The Biochemistry (content expert) lecturers (facilitator) always know how to guide us in getting to the right learning issues.”***

***“The content expert always probe us to think outside the box. Since each PBL case correlates between Biochemistry and Pathology, the (content expert) facilitator will (probe us) try to relate Biochemistry and its pathways involved in the pathogenesis of the disease.”***

***“My PBL session was once extended up to 4 hours because the (content expert) facilitator probed us until we understand the concept in Medical Biochemistry. Although it was tiring, I appreciate it a lot since the facilitator triggered out our thinking skills in correlating Biochemistry with the disease”***

### Validating the efficacy of having the content expert for PBL

PBL is a part from our current teaching learning method and is it our current practice to ask a case similar to the trigger in the PBL package. Each case is translated into an essay question that carries a maximum of 10 marks. The PBL packages will stand alone; ie there was no other teaching-learning methods such as lecture involved to cover the objectives of the subject in Medical Biochemistry. Therefore, we think by assessing the raw marks will be the best method to justify our objective; to compare the efficiency of the content expert and non-content expert in PBL. Our essay question does not involve recalling knowledge (that was reflected in objective questions) and our examination committee has vetted the essay questions according to a higher level of taxonomy bloom. Our essay questions are fragmented into reasoning/explaining therefore they are true reflection of evaluating the effectiveness of PBL as one of teaching learning methods. Therefore, by statistical analysis – we can dictate the involvement of the content expert facilitators has successfully to aid the students to answer the essay better. Thus, further validation is needed to measure the achievements of the respondents. We tracked the performance of each respondent at the end of semester exam by assessing their essay marks based on the PBL packages. The data were distributed into two groups; a group who had an experience with a content expert facilitator and another group with a non-content expert (Table-II). We also divided the groups into three different categories of CGPA; high achiever (CGPA 3.01-4.00), average (2.51-3.00) and weak students (CGPA 2.0-2.5) to avoid bias in reporting the data. This is because our curriculum is based on integrated modules, which means by getting higher CGPA, students are expected to be excelled in all of the modules that include Biochemistry as one of the subtopic. Therefore, by assessing the marks based on the essay (which 100% derived from the PBL packages) is best accounted for analysis. Their scores are relatively higher than students who had zero contact with the content experts in all three categories. This trend can be seen throughout 7 different PBL packages across three different groups of CGPA. However, not all data was seen significant especially in PBL package that involved DNA & RNA as this topic requires extra practical and PBL cases to improve students’ comprehension.

**Table-II:** Students’ performance report in both Cellular Biomolecules and Metabolism essay questions related to cases discussed in the PBL. Full marks for each essay is 10. Asterisk indicates a statistical significant difference (p<0.05) between groups of content experts and non-content experts in each category of CGPA.

PBL topics	CGPA 3.01-4.0		CGPA 2.51-3.00		CGPA 2.0-2.5	
	Content experts	Non-content experts	Content experts	Non-content experts	Content experts	Non-content experts
Urea cycle	6.5 ± 0.8	6.1 ± 0.4	6.0 ± 0.7	5.6 ± 0.4	6.4 ± 0.6*	5.4 ± 0.4
Lipolysis	8.3 ± 0.4	8.0 ± 0.3	7.1 ± 1.1	6.5 ± 0.5	6.9 ± 0.8*	4.8 ± 0.6
Purine & pyrimidine metabolism	6.4 ± 0.6	6.2 ± 0.3	7.0 ± 0.9*	6.0 ± 0.2	6.0 ± 0.7*	5.2 ± 0.2
Amino acid metabolism	8.3 ± 0.3*	6.9 ± 0.4	6.3 ± 0.3	6.0 ± 0.4	4.2 ± 0.5	3.9 ± 0.4
Integration of metabolism	6.7 ± 0.4*	5.8 ± 0.6	5.3 ± 0.5	5.0 ± 0.3	3.3 ± 0.7*	2.0 ± 0.3
Structure of protein	7.8 ± 0.4	7.3 ± 0.4	5.8 ± 0.4	5.6 ± 0.4	4.7 ± 0.6*	3.1 ± 0.4
DNA & RNA	5.0 ± 0.4	4.5 ± 0.3	3.8 ± 0.3	3.5 ± 0.3	2.5 ± 0.4	2.3 ± 0.3

* Values are reported as mean ± SD.

## DISCUSSION

### Benefits of having the content expert as a facilitator in PBL

With the experience and the precise knowledge of Medical Biochemistry, students found it necessarily helpful that the content experts function as facilitators. They are better at guiding the students in the session with enquiries that are directed towards the goal of the session. Having the expertise enables them to ask questions that make connection between what students have learned in their lectures and the given case. Hence, it facilitates better application of the knowledge they gained and maximises their knowledge retention as they are able to establish more profound connection between lectures and PBL. This is quite similar to tutoring approach of Socrates; where they seek to draw as much as possible out of their students and to make learning an active and constructive process.^8^

The content expert facilitators are more likely to encourage the students to articulate the reasoning and meaning underlying their thinking, for example by stimulating self- generated explanations, so when it comes to the second session of the PBL, they are able to evoke thoughts even better within the students during the discussion.^9^ They can contribute some of the greater perspectives of the session based on the expert’s experience such as the latest breakthrough in that field or the latest discovery and techniques, establishing connection between the session and future practices and eventually, allowing the student to appreciate the session better which indirectly leads them to increase their knowledge retention on that matter and sparks a life-long learning mood. Such notion is illustrated by a study done by Hay & Katsikitis^10^, where they have found that students being facilitated by a content expert scored higher marks in the written exam as compared to the non-expert. However, there was a concern that students with a content expert facilitator might miss opportunities to learn how to prioritise their learning needs, ask and answer crucial problem and synthesize their learning.^3^,^5^-^6^ While having content experts is undeniably commendable and has so much to offer in the real world practise^11^, it’s safe to say that such mission would be a little far-fetched. In current situation, there are less content experts in the medical faculty to cater the needs of PBL. Henceforth, the university needs to adapt, utilise and make the most of their resources and expertise in order to still maintain an effective PBL session within their curriculum.^12^-^14^

### Alternative for the lack of content experts; “Meet-the-Expert-Session

Another alternative to the PBL approach is the Meet-the-Expert-Session, where it grasps the concept of team-based learning (TBL).^15^-^16^ This approach is supplementary to the PBL, and allows limited number of content expert facilitator attend bigger amount of students per group (n=20) as compared to the PBL group (n=8 to 12). Each group is assigned to certain tasks/assignments which will be given a week prior the meeting, so they can find resources earlier before the case discussion. The main characteristics of TBL are: *(i)* professionally relevant problems, *(ii)* small self-managed teams, *(iii)* mandatory pre-class preparation by students, *(iv)* an individual and a team test to determine students’ readiness for dealing with complex decision-based professionally relevant problems, and *(v)* working on problems in teams.^17^ A combination of both PBL and TBL sets a paradigm for an encouraging learning environment as this can benefit both students and content expert by obtaining structured peer feedback and inter-team discussion during the TBL session and the pre-reading assignments, and by generating questions based on their reading through learning issues as in PBL.^6^,^18^

**Table:** SUPPLEMENTARY

Q1: Explain what do you know about PBL? How do you like PBL as a teaching-learning method in UKM?
Q2: Which PBL session do you prefer the most? Session 1 (probing to know the learning outcomes) or Session 2 (presenting the learning outcomes)?
Q3: What do you think about the effectiveness of PBL? How about the case given?
Q4: Can you tell about your experiences with your facilitators?
Q5: In your opinion, in what ways do you think that PBL can be improved?
Q6: What is the key of conducting an effective PBL? What is your perception towards facilitators as a content expert?
Q7: What is your limitation when participating in the PBL session?

### Limitations of the study

Only two cohorts were involved in this study. More samples are needed to further justify our conclusion.

## CONCLUSION

Students perceived to appreciate PBL better when having a content expert facilitator as compared to the non-content expert and this is depicted by their improved performance in the written examination.
